# Evaluation of tumour heterogeneity by ^18^F-fluoroestradiol PET as a predictive measure in breast cancer patients receiving palbociclib combined with endocrine treatment

**DOI:** 10.1186/s13058-022-01555-7

**Published:** 2022-08-26

**Authors:** Cheng Liu, Shihui Hu, Xiaoping Xu, Yongping Zhang, Biyun Wang, Shaoli Song, Zhongyi Yang

**Affiliations:** 1 Department of Nuclear Medicine, Fudan University Shanghai Cancer Center, No.270, Dong’an Road, Xuhui District, Shanghai, 200032 China; 2 Department of Medical Oncology, Fudan University Shanghai Cancer Center, Shanghai, 200032 China; 3 Department of Oncology, Shanghai Medical College, Fudan University, Shanghai, 200032 China; 4 Shanghai Institute of Medical Imaging, Fudan University, Shanghai, 200032 China; 5 Center for Biomedical Imaging, Fudan University, Shanghai, 200032 China; 6 Shanghai Engineering Research Center of Molecular Imaging Probes, Shanghai, 200032 China

**Keywords:** ^18^F-FES, Tumour heterogeneity, Palbociclib, Endocrine therapy, Metastatic breast cancer

## Abstract

**Background:**

Predictive biomarkers are needed to identify oestrogen receptor-positive, human epidermal growth factor receptor 2-negative (ER + /HER2-) metastatic breast cancer (MBC) patients who would likely benefit from cyclin-dependent kinase 4 and 6 inhibitors combined with endocrine therapy. Therefore, we performed an exploratory study to evaluate the tumour heterogeneity parameters based on 16α-^18^F-fluoro-17β-oestradiol (^18^F-FES)-PET imaging as a potential marker to predict progression-free survival (PFS) in MBC patients receiving palbociclib combined with endocrine therapy.

**Methods:**

Fifty-six ER + MBC patients underwent ^18^F-FES-PET/CT before the initiation of palbociclib. ^18^F-FES uptake was quantified and expressed as the standardized uptake value (SUV). Interlesional heterogeneity was qualitatively identified according to the presence or absence of ^18^F-FES-negative lesions. Intralesional heterogeneity was measured by the SUV-based heterogeneity index (HI = SUVmax/SUVmean). Association with survival was evaluated using the Cox proportional hazards model.

**Results:**

A total of 551 metastatic lesions were found in 56 patients: 507 lesions were identified as ^18^F-FES-positive, 38 lesions were distributed across 10 patients without ^18^F-FES uptake, and the remaining 6 were liver lesions. Forty-three patients obtained a clinical benefit, and 13 developed progressive disease (PD) within 24 weeks. Nine out of 10 patients with an ^18^F-FES-negative site developed PD, and the median PFS was only 2.4 months. Among 46 patients with only ^18^F-FES-positive lesions, only four patients had PD, and the median PFS was 23.6 months. There were statistically significant differences between the two groups (*P* < 0.001). For the subgroup of patients with only ^18^F-FES-positive lesions, low FES-HI patients experienced substantially longer PFS times than those with high FES-HI (26.5 months vs. 16.5 months, *P* = 0.004).

**Conclusions:**

^18^F-FES-PET may provide a promising method for identifying and selecting candidate ER + /HER2- MBC patients who would most likely benefit from palbociclib combined with endocrine treatment and could serve as a predictive marker for treatment response.

*Trial registration* NCT04992156, Date of registration: August 5, 2021 (retrospectively registered).

## Background

Breast cancer is the most common malignant tumour in women and a leading cause of cancer-related deaths worldwide [1]. Hormone receptor-positive (HR +) breast cancer is the most common subtype and is a candidate for endocrine therapy [2]. Administering cyclin-dependent 4/6 kinase (CDK4/6) inhibitors in combination with endocrine therapy has become a standard of care for patients with HR + /human epidermal growth factor receptor 2-negative (HR + /HER2-) metastatic breast cancer (MBC) [3–5]. Palbociclib, a first-in-class, orally administered CDK4/6 inhibitor, has been shown to exhibit antitumour activity by causing cell cycle arrest and was approved in combination with endocrine therapy to treat HR + /HER2- MBC patients [6]. However, although this combination is highly effective, the majority of patients experience disease progression during treatment, and another cohort of patients is intrinsically resistant to the combination of CDK4/6 inhibitors and endocrine therapy [7–9]. As a result, predicting the patient response to CDK4/6 inhibitors plus endocrine therapy has become an area of major scientific interest so that both side effects, such as neutropenia, leukopenia, fatigue, and nausea, and high treatment costs can be avoided. Furthermore, depending on the predicted response, aggressive treatments could be commenced in those who may benefit from the combination of CDK4/6 inhibitors and endocrine therapy, whereas other more effective treatments could be introduced at an early time point for patients who are unlikely to respond.

At present, some potentially predictive biomarkers have been found for CDK4/6 inhibitors, such as cyclin E1 (CCNE1) [10], thymidine kinase 1 (TK1) mRNA expression [11], and circulating tumour DNA (ctDNA) [12]. However, these promising biomarkers have not been adopted in clinical practice because they are neither fully predictive nor routinely available. CDK4/6 enzymes are key promoters of tumour growth in HR + breast cancer and cooperate with oestrogen receptor (ER) pathway activation [13, 14]. ER status is essential in the selection of treatment protocols and is a well-known prognostic factor [5, 15, 16]. Moreover, higher ER expression is often associated with a better outcome for endocrine therapy [17]. Quite a few ER-positive primary breast cancer patients may eventually develop ER-negative metastatic lesions, and these patients are unlikely to benefit from ER-directed therapies [18, 19]. Collecting biopsy samples from metastatic tissue is not always feasible in daily practice due to the location of the metastatic lesion and the risks associated with biopsy [20]. In addition, a single biopsy may not be representative of the entire lesion, and tumour heterogeneity may limit the validity of the assessment of a single lesion [21].

Positron emission tomography (PET) with 16α-^18^F-fluoro-17β-oestradiol (^18^F-FES) is a noninvasive method that visualizes and quantifies the expression of ER in multiple tumours throughout the body (excluding lesions in the liver, where it is metabolized) [22, 23]. Other researchers and our previous studies have shown that ^18^F-FES uptake correlated well with ER expression measured by immunohistochemical staining, and ^18^F-FES PET played an important role in predicting the response to endocrine therapy [24–29]. Tumour heterogeneity has been shown to have a profound impact on malignant behaviour and treatment response, and molecular imaging provides an important noninvasive method for biologically characterizing tumour heterogeneity and predicting treatment results. Therefore, our study specifically aimed to assess the heterogeneity of intralesional and interlesional ER expression, as measured by ^18^F-FES-PET, to identify patients who may benefit from combination therapy and provide early predictive factors.

## Methods

### Patients

This retrospective study was conducted in accordance with the principles of Good Clinical Practice and the Declaration of Helsinki. The requirement for informed consent was waived owing to the retrospective nature of the study. This study was retrospectively registered with ClinicalTrials.gov (ClinicalTrials.gov identifier: NCT04992156), and the other study ID number is YOUNGBC-15.

The subjects were patients with HR + /HER2- MBC who initiated palbociclib plus endocrine therapy between March 2017 and December 2020 in the Fudan University Shanghai Cancer Center. Patients who underwent an ^18^F-FES PET/computed tomography (CT) scan before the first regimen were enrolled in this study. Additional inclusion criteria were as follows: (1) female sex; (2) age ≥ 18 years; (3) histologically and cytologically confirmed MBC; (4) HR-positive and HER2-negative status, as defined according to the American Society of Clinical Oncology (ASCO)/College of American Pathologists (CAP) guidelines; and (5) complete medical records.

### Assessment of treatment response

The patients underwent CT or magnetic resonance imaging (MRI) every 2–3 months during treatment until disease progression. Tumour response was assessed by the attending physicians according to the Response Evaluation Criteria in Solid Tumours (RECIST) 1.1. Clinical data regarding baseline patient characteristics, treatment history, and efficacy of palbociclib plus endocrine therapy were retrospectively acquired from the electronic medical record system.

The primary endpoint of this study was progression-free survival (PFS), which was defined as the time from treatment initiation to disease progression or death from any cause. The second objective of this study was to evaluate the clinical benefit rate (CBR), which was defined as the percentage of patients experiencing complete response (CR), partial response (PR), and stable disease (SD) for at least 24 weeks according to the RECIST 1.1 criteria.

### ^***18***^***F-FES PET/CT procedure***

The synthesis and quality control of ^18^F-FES were conducted as described previously [27, 30]. The patients received approximately 222 MBq of ^18^F-FES intravenously over 1–2 min. Whole-body (head to mid-thigh) PET/CT was performed 60 min after tracer injection using a Siemens Biograph 16 HR PET/CT scanner or mCT Flow PET/CT scanner (Knoxville, Tennessee, USA) according to European Association of Nuclear Medicine (EANM) guidelines [31]. Low-dose CT was acquired for attenuation and scatter correction. A PET emission scan covering the same spatial range was performed immediately after the low-dose CT scan. To analyse the images, Gaussian filter iteration was used to reconstruct the emission images. Patients who had been administered ER antagonists were required to discontinue them for at least 6 weeks to avoid false-negative ^18^F-FES results [29].

### Image analysis

We used a multimodality computer platform (Syngo, Siemens, Knoxville, TN, USA) for image viewing and tracer uptake quantification. Two board-certified nuclear medicine physicians (> 5 years of working experience) evaluated the images independently and were blinded to the clinical outcomes. In the case of a discrepancy between the two physicians, a consensus was reached on a final reading for the statistical analyses. Volumes of interest (VOIs) were manually drawn around the area of avid tumour uptake visible on PET (higher than adjacent normal tissue background) with the corresponding low-dose CT serving as a guide. The lesions outlined on the ^18^F-FES PET image have to be identified and located by ^18^F-FDG PET/CT and diagnostic CT or MRI. To reduce the partial volume effect and the limitation of resolution, ^18^F-FES uptake was quantitated in measurable lesions with diameters greater than 1.0 cm. In patients with numerous metastatic lesions, up to an arbitrary maximum of 20 lesions were selected for analysis according to the guidelines of the EANM [31].

The ^18^F-FES uptake of a lesion was semiquantitatively expressed as the maximum standardized uptake value (SUVmax) [31]. The mean standardized uptake value (SUVmean) was quantified using a 50% threshold of the SUVmax of the lesion. In line with previous studies in our centre, those with SUVmax ≥ 1.8 were defined as FES-positive lesions [27]. FES-Hot5 was defined as the geometric mean FES SUVmax of the 5 hottest lesions (up to 5 lesions per patient). Interlesional heterogeneity was qualitatively identified according to whether the patient had or did not have FES-negative lesions. A quantitative measure of intralesional heterogeneity, the heterogeneity index (HI), which has been previously used in breast cancer patients, was obtained by dividing the SUVmax by the SUVmean (HI = SUVmax/SUVmean) [27, 32].

### Statistical analysis

Continuous variables are displayed as the median value and range. Survival analyses were performed using the Kaplan‒Meier method, and survival was compared by the log-rank test. Each parameter was dichotomized using the median as a threshold. The Cox proportional hazards model was used for univariate and multivariate analyses, and the results are expressed as the hazard ratio with its corresponding 95% confidence interval [CI] and *P* value. Multivariate analysis with forward stepwise selection was performed with the variables that were proven to be significant in univariate analysis to explore independent significant factors. The association between pretreatment ^18^F-FES PET image parameters and patients with a clinical benefit from palbociclib combined with endocrine therapy was calculated by the Pearson’s chi-square test or Mann‒Whitney U test. All data analyses were performed using SPSS Statistics version 20.0 (IBM Corporation, Armonk, NY, USA). All statistical tests were two-sided, and a *P* value less than 0.05 denoted a statistically significant difference.

## Results

### Patient characteristics

**Fig. 1:**
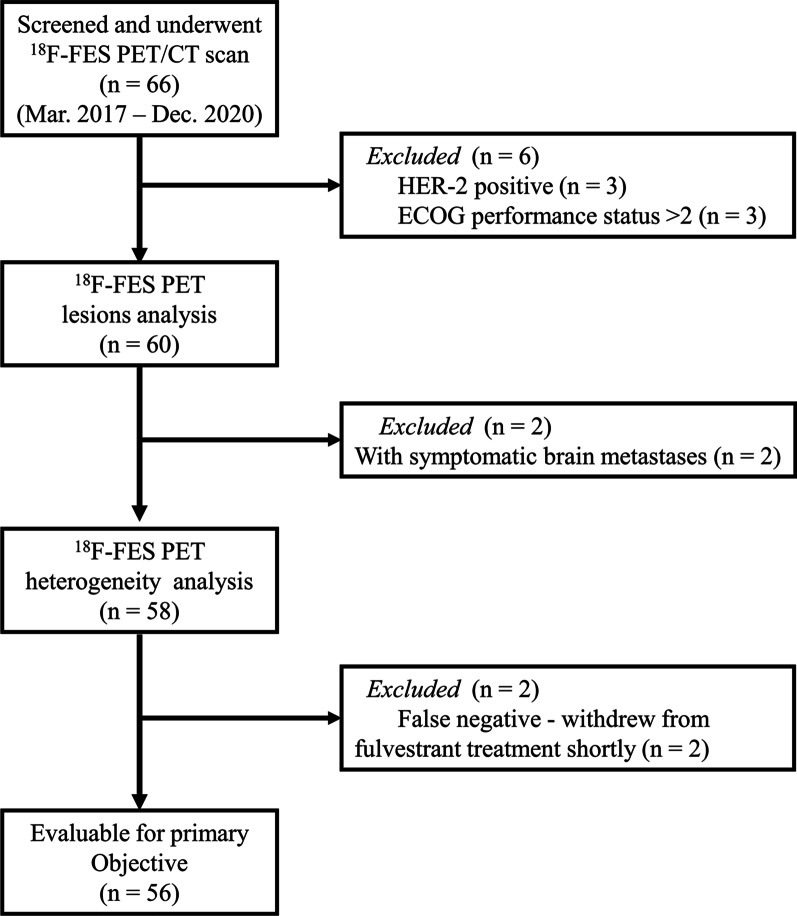
Patient flowchart for inclusion and exclusion. Abbreviations: *HER-2*, human epidermal growth receptor 2; *ECOG*, Eastern Cooperative Oncology Group

From March 2017 to December 2020, 66 patients with MBC underwent an ^18^F-FES PET/CT scan before starting treatment with palbociclib. A total of 10 patients were excluded from this analysis: three had HER2 + disease, three had an Eastern Cooperative Oncology Group (ECOG) performance status score of > 2, and two had symptomatic brain metastases. Furthermore, in two patients, the treatment response to fulvestrant was evaluated by ^18^F-FES PET/CT, and the drug was not discontinued before the examination. The lesions of both patients were ^18^F-FES negative, which we considered to be false negatives [33], so they were excluded from the final analysis. After excluding 10 patients who were not eligible, a total of 56 patients were eventually included in our analysis, as illustrated in Fig. 1.

Of these 56 patients, the median age was 55.5 years (range, 23–74 years), 42 patients had a natural postmenopausal status, 14 patients achieved postmenopausal status by the use of luteinizing hormone releasing hormone (LHRH) agonists, 37 patients received palbociclib combined with fulvestrant, and 19 patients received palbociclib plus letrozole. The numbers of patients receiving palbociclib in the first-, second-, third-, and later-line settings were 38 (67.8%), 9 (16.1%), 4 (7.2%), and 5 (8.9%), respectively. Nearly one-third of the patients (32.1%) had at least three metastatic sites, with bone being the most common metastatic site (66.1%), and nearly one-half of the patients (46.4%) had visceral metastasis. The clinical characteristics of the 56 MBC patients are listed in Table 1.

**Table 1:** Patient demographics and disease characteristics at time of FES PET scan

Characteristics	*N* = 56	%
Age, years		
Median	55.5	
Range	23–74	
< 55 years	25	44.6
≥ 55 years	31	55.4
Menopausal status		
Premenopausal ^a^	14	25.0
Postmenopausal	42	75.0
Disease-free interval ^b^		
> 5 y	31	55.4
≤ 5 y	22	39.3
Histology of primary breast cancer		
IDC	49	87.5
ILC	7	12.5
Hormone-receptor status		
ER-positive and PR-positive	46	82.1
ER-positive and PR-negative	10	17.9
Metastatic sites		
Nonvisceral	30	53.6
Bone	37	66.1
Bone-only	12	21.4
Visceral disease	26	46.4
Any lung	13	23.2
Pleural	7	12.5
Peritoneum	1	1.8
Ovarian	2	3.6
Liver	6	7.0
No. of disease sites		
1	18	32.1
2	20	35.8
≥ 3	18	32.1
De novo metastatic disease	3	5.4
Lines of therapy prior to palbociclib		
0	38	67.9
1	9	16.1
2	4	7.1
≥ 3	5	8.9
Prior ET for metastatic disease		
None	43	76.8
Yes	13	23.2
Prior ET type for metastatic disease		
Antiestrogen ± LH-RH analog	8	14.3
Aromatase inhibitor ± LH-RH analog	9	16.1
Prior chemotherapy for metastatic disease		
None	43	76.8
Yes	13	23.2
Endocrine therapy following FES PET		
palbociclib + Aromatase inhibitor	19	33.9
palbociclib + fulvestrant	37	66.1
Outcome		
CR	3	5.4
PR	6	10.7
SD	34	60.7
PD	13	23.2
Clinical benefit		
None	13	23.2
Yes	43	76.8
PFS		
Events	34	60.7
Censored	22	39.3
With negative ^18^F-FES lesions		
None	46	82.1
Yes	10	17.9

^a^ For premenopausal women, palbociclib combination with endocrine therapy was given upon the administration of gonadotropin-releasing hormone agonist

^b^ Patients with stage IV breast cancer at initial diagnosis were excluded (*N* = 3)

Abbreviations: IDC, invasive ductal carcinoma; ILC, invasive lobular carcinoma; ER, estrogen receptor; PR, progesterone receptor; ET, endocrine therapy; CR, complete responses; PR, partial responses; SD, stable disease; PD, progressive disease; PFS, progression-free survival

### ***Qualitative and quantitative results of ***^***18***^***F-FES PET/CT***

**Fig. 2:**
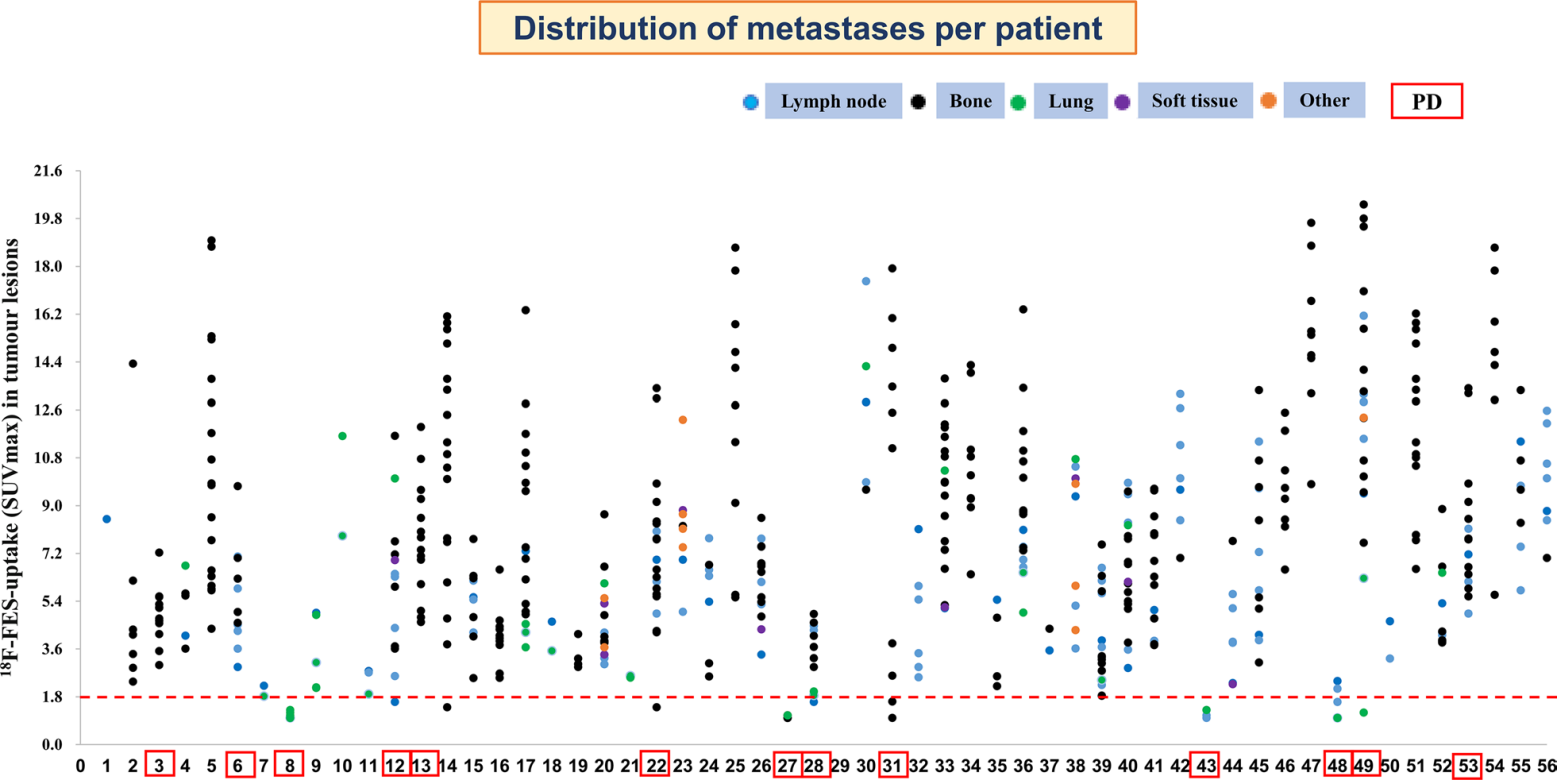
Distribution of metastases per patient and ^18^F-FES uptake (SUVmax) of all metastases in individual patients. The red dashed line indicates the SUVmax threshold of 1.8. Abbreviation: *PD*, progressive disease

A total of 551 lesions were identified and localized in 56 patients using ^18^F-FES PET/CT, ^18^F-FDG (*n* = 22), or other conventional imaging techniques. The number of lesions per patient ranged from 1 to 20, with a median of 9 lesions. Lesions were present in the bones (*n* = 361, 65.5%), lymph nodes (*n* = 127, 23.0%), lung (*n* = 26, 4.7%), pleura (*n* = 13, 2.4%), peritoneum (*n* = 1, 0.2%), ovaries (*n* = 3, 0.5%), soft tissue (*n* = 8, 1.5%), breast (*n* = 6, 1.1%), and liver (*n* = 6, 1.1%). Among 551 lesions, 507 with ^18^F-FES PET SUVmax ≥ 1.8 were identified as ^18^F-FES-positive lesions. Thirty-eight lesions (22 bone, six lymph nodes, seven lung, two soft tissue, and one breast) were considered ^18^F-FES-negative lesions in 10 patients (17.9%), out of which seven (12.5%) had both ^18^F-FES-positive and ^18^F-FES-negative lesions, and three had only ^18^F-FES-negative lesions. The other six were liver metastases, and the status of ^18^F-FES could not be quantified or qualified. The ^18^F-FES PET SUVmax varied greatly among lesions (median, 6.7; range, 1.0–20.3) and patients (median, 6.5; range, 1.1–15.4), as depicted in Fig. 2.

### ***Correlation between tumour response and ***^***18***^***F-FES PET***

Twenty patients had measurable lesions on baseline CT or MRI according to RECIST, 25 patients had non-measurable visceral lesions or lymph nodes, and 12 patients had only bone metastases. At the time of analysis, 34 (60.7%) patients had documented disease progression: 33 patients developed radiological PD, and one patient developed deterioration of symptoms and a fourfold increase in the tumour marker CA153, which was defined as clinical PD. An overall CBR of 76.8% was observed, with three patients with CR (5.4%), six patients with PR (10.7%) and 34 patients with SD (60.7%) at ≥ 24 weeks. A total of 13 patients (23.2%) had PD within 6 months from palbociclib combined with endocrine therapy.

The baseline tumour ^18^F-FES uptake in metastatic patients with clinical benefit from palbociclib was similar to that in patients with PD (median SUV max, 6.5 *vs*. 5.0; *P* > 0.05). However, it is interesting that nine of 10 patients with at least one ^18^F-FES-negative site developed PD, and among 46 patients with 100% ^18^F-FES-positive disease, only four patients had PD within 6 months (Fig. 2, *P* < 0.001). Using the presence of any ^18^F-FES-negative metastatic lesion to distinguish between patients with clinical benefit and PD leads to a positive predictive value (PPV) and negative predictive value (NPV) of 91.3% and 90.0%, respectively. This underlined that patients with any lesion lacking ^18^F-FES uptake above background were unlikely to benefit from palbociclib-based therapy.

### ***Predictive value of ***^***18***^***F-FES-PET for PFS***

At the time of analysis, the median PFS in the whole population was 16.1 months (range 1.9–35.6 + ; 95% CI 6.8–25.4). Before analysing ^18^F-FES PET parameters to predict PFS, we evaluated the patients’ disease characteristics before palbociclib treatment. The median PFS was 21.6 months in patients treated with palbociclib as the first line of treatment, 23.6 months as the second line of treatment, and 4.2 months as the third or further line of treatment (log-rank test *P* = 0.005). Patients treated with palbociclib as first-line and second-line treatment showed HRs of 0.33 (95% CI 0.12–0.93) and 0.29 (95% CI 0.13–0.65) for PFS compared to those treated with palbociclib as third-line or subsequent-line treatment. Nevertheless, the disease-free interval (DFI) from adjuvant treatment, number of disease sites, presence of visceral disease and types of endocrine therapy were not found to be prognostic factors of PFS in the whole cohort (Table 2).

**Table 2:** Univariate and multivariate Cox regression analyses for prediction of PFS for the entire patients

Parameters	No	Event	Median PFS	Log-rank	Univariate analysis		Multivariate analysis	
			(95% CI)	*P* value	HR (95% CI)	*P* value	HR (95% CI)	*P* value
*Disease-free interval*								
≤ 5 y	22	11	23.9(1.6–46.3)	0.366	1.40(0.67–2.89)	0.369	NA	
> 5 y	31	22	15.6(10.1–21.1)					
*No. of disease sites*								
1	18	13	12.0(0.9–23.1)	0.690				
2	20	12	12.1(0.6–23.6)		0.89(0.41–1.97)	0.789	NA	
≥ 3	18	9	23.9(14.6–33.2)		0.69(0.29–1.63)	0.397		
*Visceral disease*								
No	30	20	12.0(6.4–17.6)	0.191	0.64(0.32–1.26)	0.196	NA	
Yes	26	14	23.9(14.4–33.3)					
*Lines of therapy prior to palbociclib*								
0	38	19	21.6(12.6–30.6)		0.33(0.12–0.93)	0.036*		
1	9	6	23.6(7.8–39.4)	0.005*	0.29(0.13–0.65)	0.003*	/	0.170
≥ 2	9	9	4.2(3.8–4.7)					
*Types of endocrine therapy*								
palbociclib + fulvestrant	37	25	12.8(7.0–18.7)	0.186	0.60(0.28–1.29)	0.192	NA	
palbociclib + letrozole	19	9	26.5(4.5.7–48.5)					
*Presence of FES-negative lesions*								
Yes	10	10	2.4(1.1–3.7)	< 0.001*	0.04(0.01–0.13)	< 0.001*	0.04(0.01–0.13)	< 0.001*
No	46	24	23.6(15.8–31.4)					

*PFS* Progression-free survival; *CI* Confidence interval; *HR* Hazard ratio; *MBC* Metastatic breast cancer; *SUVmax* Maximum standard uptake value

^*^ Indicates statistically significant differences (*P* < 0.05); N/A: Analysis not performed as univariate analysis not significant

**Fig. 3:**
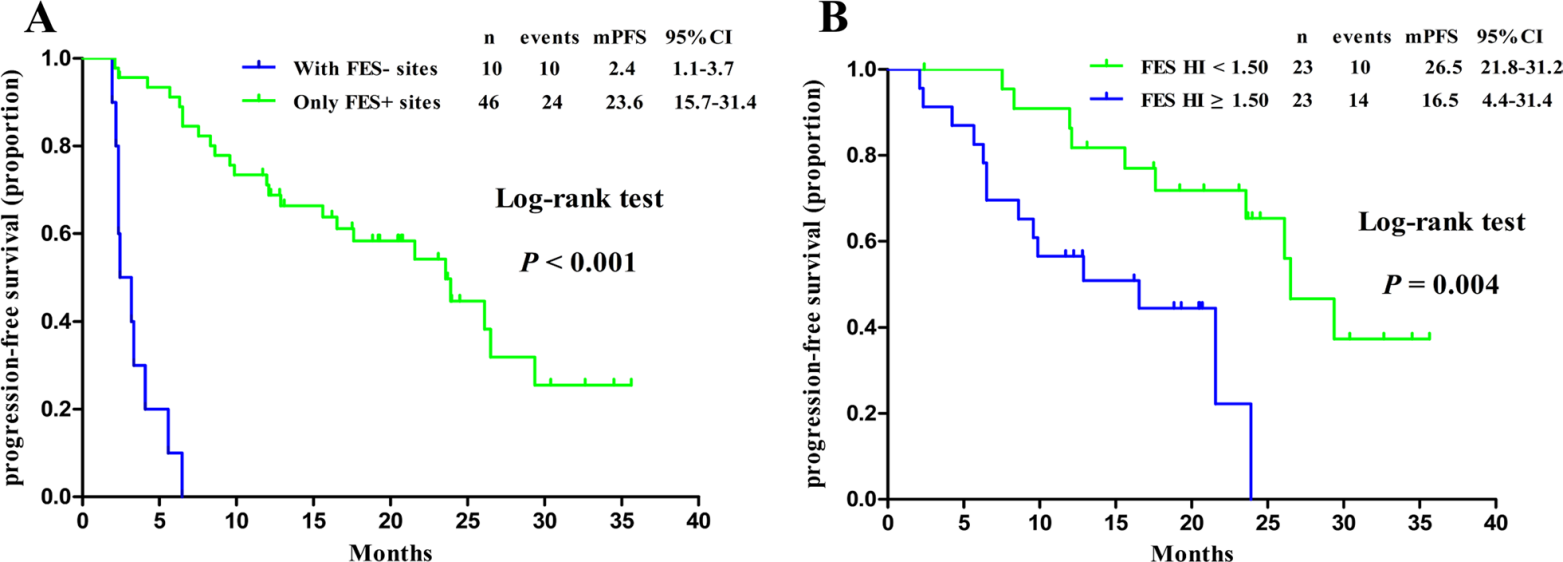
Kaplan‒Meier curve of progression-free survival (PFS) according to heterogeneity determined by ^18^F-FES PET. ** A** PFS predicted by interlesional heterogeneity, with patients stratified by the presence or absence of ^18^F-FES-negative lesions in the whole cohort. ** B** PFS predicted by intralesional heterogeneity, with patients stratified by the median FES-HI in the subgroup cohort with only ^18^F-FES-positive lesions

**Fig. 4:**
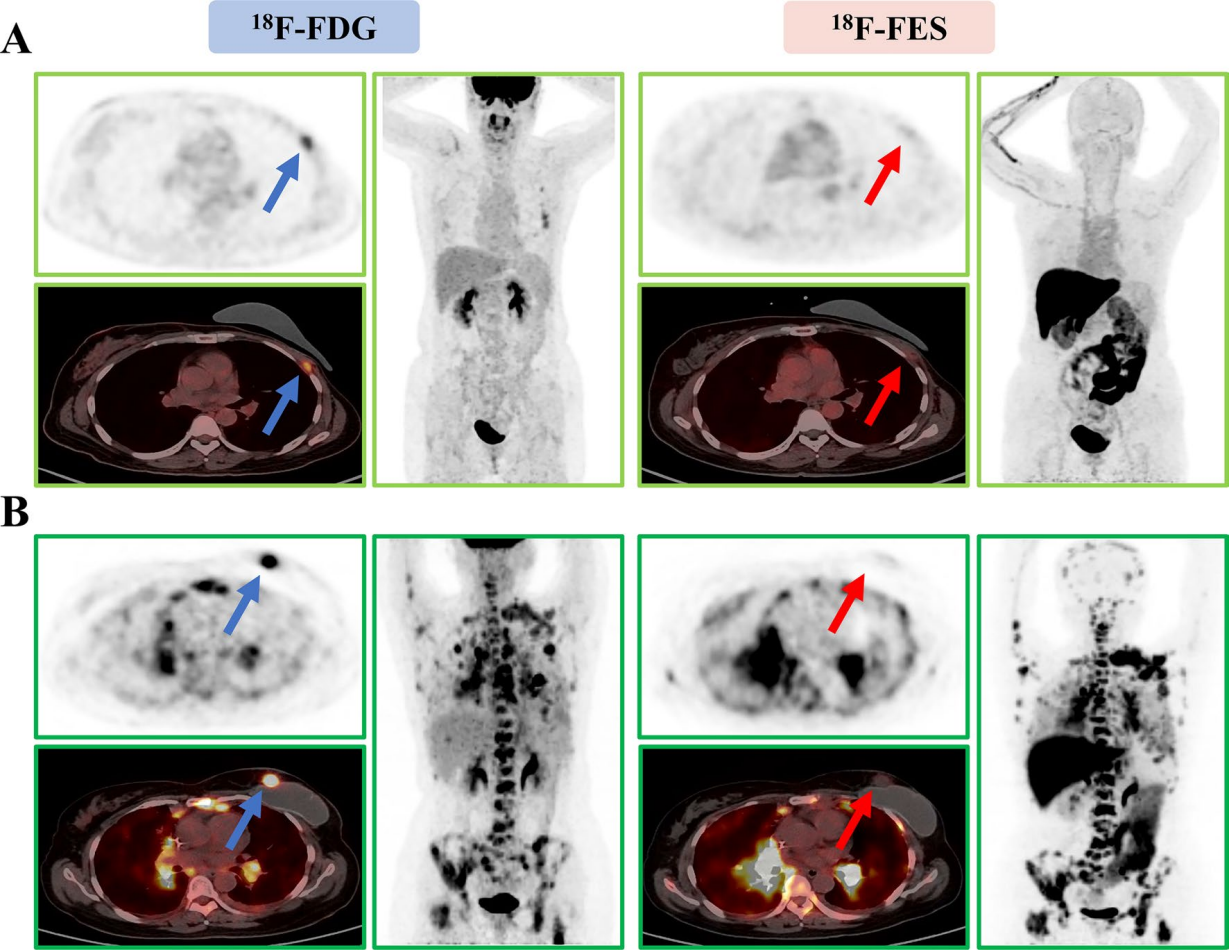
Representative imaging of patients with at least one ^18^F-FES-negative lesion. **A** Only ^18^F-FES-negative lesions (Fig. 2, #43). This 50-year-old woman had 4 ^18^F-FDG-positive lesions in her chest wall and lymph nodes, but all were negative on ^18^F-FES PET. She was on palbociclib combined with letrozole as first-line treatment for 2.3 months until progression occurred. ** B** Presence of ^18^F-FES-positive and ^18^F-FES-negative lesions (Fig. 2, #49). This 53-year-old woman had innumerable ^18^F-FDG-positive and ^18^F-FES-positive lesions in the pleura, lung, lymph nodes, and bone, but the left chest wall metastasis showed outstanding uptake of ^18^F-FDG but not of ^18^F-FES. She was on palbociclib combined with fulvestrant as third-line treatment for 5.6 months until progression occurred

Next, we evaluated the predictive value of ^18^F-FES PET parameters for survival in patients receiving palbociclib-based therapy. A total of 46 patients had only ^18^F-FES-positive sites, and their median PFS was 23.6 months (95% CI 15.8–31.4); 10 patients (seven had both positive and negative ^18^F-FES metastases, and three had no ^18^F-FES-positive metastases) had at least one ^18^F-FES-negative metastatic site, and their median PFS was only 2.4 months (95% CI 1.1–3.7) (log-rank *P* < 0.001, Fig. 3A and Fig. 4). Using univariate analysis, we found lines of advanced systemic therapy with palbociclib and presence of ^18^F-FES-negative lesions to be significantly correlated with PFS. However, in multivariate analysis using Cox proportional hazards models, only presence of ^18^F-FES-negative lesions was found to be a single determinant of PFS (HR = 0.04, 95% CI 0.01–0.13, *P* < 0.001, Table 2).

**Fig. 5:**
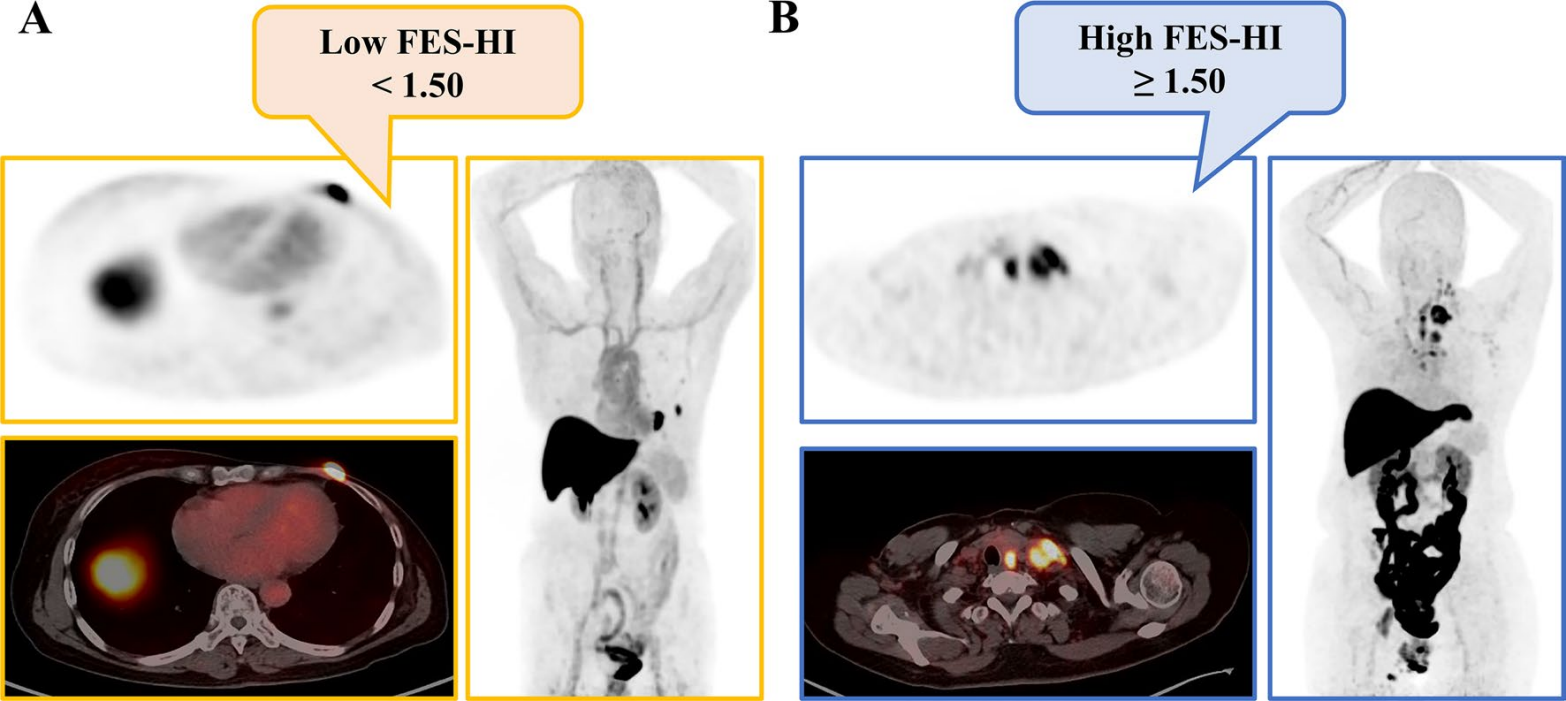
Representative imaging of patients with only ^18^F-FES-positive lesions. ** A** Low FES-HI (Fig. 2, #10). This 67-year-old woman had 2 ^18^F-FES-positive lesions in the chest wall and lung, with no ^18^F-FES-negative lesions. The median FES-HI for the 2 avid lesions was 1.38. She was on palbociclib combined with letrozole as first-line treatment for 30.4 months without disease progression. **B** High FES-HI (Fig. 2, #32). This 56-year-old woman had 6 ^18^F-FES-positive lesions in the lymph nodes, with no ^18^F-FES-negative lesions. The median FES-HI for the 6 avid lesions was 1.52. She was on palbociclib combined with fulvestrant as first-line treatment for 16.5 months until progression

The presence or absence of ^18^F-FES-negative lesions represents only interlesional heterogeneity and not intralesional heterogeneity. Hence, we conducted an exploratory analysis using identified PET biomarkers to predict survival in patients with only ^18^F-FES-positive lesions. Intralesional heterogeneity was measured by dividing the SUVmax by the SUVmean across the ^18^F-FES-positive metastatic lesions, which is the HI. In the ^18^F-FES-positive subgroup analysis, the median values of SUVmax, FES-Hot5 and HI were used as the cut-off values, which were 6.5 (range 1.9–15.4), 8.1 (range 2.0–17.2) and 1.50 (range 1.33–1.57), respectively. Regrettably, SUVmax and FES-Hot5 were not predictive of PFS in this subgroup (log-rank *P* = 0.258 and 0.575, respectively, Table 3). A total of 23 patients with high HI-FES had obviously shorter PFS times than those with low HI (HI ≥ 1.50, median PFS 16.5 months, 95% CI 4.4–28.6 *vs.* HI < 1.5, median PFS 26.5 months, 95% CI 21.8–32.2; log-rank *P* = 0.004, Fig. 3B and Fig. 5). Patients with low HI-FES showed an HR of 0.27 for PFS (95% CI 0.10–0.70; *P* = 0.007) compared to those with high HI-FES (Table 3).

**Table 3:** Univariate and multivariate Cox regression analyses for prediction of PFS in the subgroup of patients with only FES-positive sites

Parameters	No	Event	Median PFS	Log-rank	Univariate analysis		Multivariate analysis	
			(95% CI)	*P* value	HR (95% CI)	*P* value	HR (95% CI)	*P* value
*FES SUVmax*								
≥ 6.5	23	14	21.6(13.5–29.6)	0.258	0.63(0.28–1.42)	0.262	NA	
< 6.5	23	10	29.4(13.4–45.4)					
*FES Hot5 lesions*								
≥ 8.1	23	13	23.6(14.3–32.8)	0.575	0.79(0.35–1.79)	0.576	NA	
< 8.1	23	11	23.9(9.3–38.5)					
*FES HI *^*a*^								
≥ 1.50	23	14	16.5(4.4–28.6)	0.004*	0.27(0.10–0.70)	0.007*	NA	
< 1.50	23	10	26.5(21.8–32.2)					

*PFS* Progression-free survival; *CI* Confidence interval; *HR* Hazard ratio; *SUVmax* Maximum standard uptake value; *SUVmean* Mean standard uptake value; *HI* Heterogeneity index

^a^ HI = SUVmax/SUVmean; * Indicates statistically significant differences (*P* < 0.05)

## Discussion

Currently, there are no clinically available biomarkers for prescribing CDK4/6 inhibitors except for ER expression mainly from primary tumour tissues [9, 15]. However, the expression status of ER in breast cancer may change during the course of disease progression or treatment [34]. ER status discordance rates between primary and metastatic breast cancer sites may reach approximately 32%, which might change the therapeutic strategy and sensitivity for breast cancer patients [35]. Although biopsy is the gold standard for assessing ER status, it is sometimes unreliable due to interlesional and intralesional heterogeneities in ER expression. Furthermore, biopsy is an invasive procedure with the risk of serious complications and poor manoeuvrability and patient compliance. ^18^F-FES PET/CT has been shown to have the potential to assess ER expression in all tumour lesions, supporting individualized treatment strategy choices, and could aid physicians in making therapeutic decisions [30, 36].

In the present study, we investigated the ^18^F-FES-PET imaging characteristics and tumour responses of patients with MBC who received a CDK4/6 inhibitor combined with endocrine therapy. Boers et al. found that patients with 100% ^18^F-FES positivity benefitted most from palbociclib plus letrozole compared to those with heterogeneous or absent ^18^F-FES uptake (HR 2.1) [37]. However, the limitation of this study is that it only evaluated the interlesional heterogeneity between MBC patients with and without ^18^F-FES-negative lesions and failed to investigate the intralesional heterogeneity among patients with 100% ^18^F-FES-positive metastatic lesions. Recently, a feasible quantitative method for measuring heterogeneity, HI, has been investigated. Our previous series of studies successfully used pretreatment ^18^F-FDG HI to predict survival in MBC patients [32, 38, 39], and ^18^F-FES HI can better reflect the heterogeneity of ER expression, especially in patients with metastases after treatment [27]. In this context, we designed this exploratory study to demonstrate how interlesional heterogeneity in the presence or absence of ER-positive lesions and intralesional heterogeneity in all ER-positive patients could predict the efficacy of palbociclib plus letrozole or fulvestrant based on ^18^F-FES-PET imaging.

Our study demonstrated that pretreatment ^18^F-FES PET has value in predicting whether patients treated with palbociclib will respond to therapy. From our data, patients (9/10) who had any ^18^F-FES-negative lesions were more likely to develop PD within 24 weeks of therapy initiation (with no clinical benefit). In contrast, almost all patients (42/46) who had only ^18^F-FES-positive lesions obtained a clinical benefit. This is different from the results of Boers et al*.*, who found that a considerable number of ^18^F-FES-negative lesions also showed a response [37]. One explanation may be that some of their ^18^F-FES-negative lesions may still exhibit mild ER expression because they used an ^18^F-FES SUVmax cut-off of 2.0, which is higher than the value of 1.8 we used. Another explanation could be the differences in treatment patterns and patient characteristics, and the efficacy of palbociclib in the real-world setting differed. However, based on their research, it seems that physicians will still be puzzled by whether the presence of ^18^F-FES-negative lesions means that patients can benefit from palbociclib plus endocrine therapy. In comparison, our imaging indicators provided a better-stratified method to identify who could benefit from palbociclib plus endocrine therapy and who is not likely to benefit, which is more in conformity with the concept of precision medicine.

In the survival analysis, patients with ^18^F-FES-negative lesions exhibited a poorer prognosis with an obviously shorter median PFS than those who had only ^18^F-FES-positive lesions (2.4 months vs. 23.6 months, log-rank *P* < 0.001). Moreover, patients with additional lines of advanced systemic therapy with palbociclib (*P* = 0.005) had a worse prognosis. However, due to the strong interplay between ER pathways and CDK4/6 signalling, only the presence or absence of ^18^F-FES uptake significantly and independently correlated with the outcome in the multivariate analysis. Moreover, one specific difference between our report and previous reports is that we further analysed the subgroup of patients with only ^18^F-FES-positive lesions. This was undertaken because, even if this cohort has a good response to palbociclib treatment, considering the heterogeneity of ER expression in tumours, the final efficacy in each patient may be different. As expected, patients with a low FES-HI had significantly longer PFS times than those with a high FES-HI (median PFS, 26.5 months vs. 16.5 months, log-rank *P* = 0.004).

Thus, our results suggested that for ER-positive (primary) MBC, patients with any ER-negative lesion by ^18^F-FES PET are unlikely to benefit from palbociclib plus endocrine therapy, and it might be better to make changes to the treatment protocol. Patients with only ^18^F-FES-positive lesions are potential candidates for combination therapy, but the efficacy in patients with high FES-HI is unsatisfactory. This group of patients should choose chemotherapy or other endocrine therapy options, such as chidamide in combination with endocrine therapy or another CDK4/6 inhibitor in combination with endocrine therapy [40].

There were some limitations to this study. First, given the retrospective design of the study, the disease characteristics of the patients in the cohort were heterogeneous, which may include patients with inherently different prognostic factors independent of ^18^F-FES uptake. However, we eliminated some known factors that can affect ^18^F-FES uptake, such as discontinuation of drugs known to bind ER less than 6 weeks before ^18^F-FES PET imaging. In addition, the study was conducted in a single centre and was based on a small cohort of Asian patients, so the optimal cut-off values identified in this study might not be applicable to all patients, and external validation is needed. Given that the patients in our study underwent many pretreatments, but only some patients had an ^18^F-FDG PET scan concurrently, CT or MRI scans may have shown bone lesions that were no longer active, leading to an overestimation of the number of ^18^F-FES-negative lesions. Moreover, our study did not analyse the relationship between ^18^F-FES uptake and ESR1 gene amplification and mutation in the tumour biopsy, as these assays provide insufficient data at present to guide therapy for HR + /HER2- MBC[41] and are not performed routinely in our centre. Although ER expression is required for a response to palbociclib plus endocrine therapy, other pathways may also affect the efficacy of palbociclib, such as the androgen receptor (AR) signalling pathway [42]. A single ^18^F-FES PET scan failed to show crosstalk with other pathways; therefore, it would be of interest in future studies to add multiple molecular imaging probes to improve the predicted response to palbociclib-based treatment, such as ^18^F-fluoro-5α-dihydrotestosterone (^18^F-FDHT)-PET for imaging AR [43] and ^18^F-FDG PET for imaging the glycolytic metabolism tumour burden [44]. Finally, we were unable to obtain serial tumour biopsies to assess ER status, especially liver metastases (ER status cannot be reliably measured in liver metastases due to high background ^18^F-FES avidity), thus limiting information on the accuracy of the ^18^F-FES PET imaging of ER status.

## Conclusion

Our study showed that interlesional and intralesional heterogeneity demonstrated by ^18^F-FES-PET/CT provided a promising way to predict palbociclib plus endocrine therapy efficacy and provided a novel method for better stratifying and selecting candidate MBC patients who would most likely benefit from palbociclib plus endocrine therapy.

## Data Availability

The datasets used and analysed during the current study are available from the corresponding author on reasonable request.

## Abbreviations

ER + /HER2-: Estrogen receptor positive, human epidermal growth factor receptor 2 negative
MBC: Metastatic breast cancer
CDK4/6: Cyclin-dependent kinase 4 and 6
^18^F-FES: 16α-^18^F-fluoro-17β-oestradiol
PET: Positron emission tomography
HI: Heterogeneity index
PFS: Progression-free survival
SUV: Standardized uptake value
HI: Heterogeneity index
PD: Progressive disease
HR +: Hormone receptor-positive
ECOG: Eastern cooperative oncology group
AI: Aromatase inhibitor
SERD: Selective estrogen receptor degrader
LHRHa: Luteinizing hormone-releasing hormone analogue
PPV: Positive predictive value
NPV: Negative predictive value
CBR: Clinical benefit rate
CR: Complete response
PR: Partial response
SD: Stable disease
VOIs: Volumes of interest
HR: Hazard ratio
CI: Confidence interval
^18^F-FDG: ^18^F-fuorodeoxyglucose
AR: Androgen receptor
^18^F-FDHT: ^18^F-fluoro-5α-dihydrotestosterone
